# Symmetry Breaking Charge Transfer in DNA-Templated Perylene Dimer Aggregates

**DOI:** 10.3390/molecules27196612

**Published:** 2022-10-05

**Authors:** Katelyn M. Duncan, Donald L. Kellis, Jonathan S. Huff, Matthew S. Barclay, Jeunghoon Lee, Daniel B. Turner, Paul H. Davis, Bernard Yurke, William B. Knowlton, Ryan D. Pensack

**Affiliations:** 1 Micron School of Materials Science & Engineering, Boise State University, Boise, ID 83725, USA; 2 Department of Chemistry & Biochemistry, Boise State University, Boise, ID 83725, USA; 3 Center for Advanced Energy Studies, Idaho Falls, ID 83401, USA; 4 Department of Electrical & Computer Engineering, Boise State University, Boise, ID 83725, USA

**Keywords:** perylene, dimer aggregate, DNA nanotechnology, singlet fission, charge transfer

## Abstract

Molecular aggregates are of interest to a broad range of fields including light harvesting, organic optoelectronics, and nanoscale computing. In molecular aggregates, nonradiative decay pathways may emerge that were not present in the constituent molecules. Such nonradiative decay pathways may include singlet fission, excimer relaxation, and symmetry-breaking charge transfer. Singlet fission, sometimes referred to as excitation multiplication, is of great interest to the fields of energy conversion and quantum information. For example, endothermic singlet fission, which avoids energy loss, has been observed in covalently bound, linear perylene trimers and tetramers. In this work, the electronic structure and excited-state dynamics of dimers of a perylene derivative templated using DNA were investigated. Specifically, DNA Holliday junctions were used to template the aggregation of two perylene molecules covalently linked to a modified uracil nucleobase through an ethynyl group. The perylenes were templated in the form of monomer, transverse dimer, and adjacent dimer configurations. The electronic structure of the perylene monomers and dimers were characterized via steady-state absorption and fluorescence spectroscopy. Initial insights into their excited-state dynamics were gleaned from relative fluorescence intensity measurements, which indicated that a new nonradiative decay pathway emerges in the dimers. Femtosecond visible transient absorption spectroscopy was subsequently used to elucidate the excited-state dynamics. A new excited-state absorption feature grows in on the tens of picosecond timescale in the dimers, which is attributed to the formation of perylene anions and cations resulting from symmetry-breaking charge transfer. Given the close proximity required for symmetry-breaking charge transfer, the results shed promising light on the prospect of singlet fission in DNA-templated molecular aggregates.

## 1. Introduction

Singlet fission is a process in aggregates of conjugated organic molecules that has relevance in fields ranging from solar energy conversion [1,2,3] to quantum information science [4,5]. In singlet fission, one overall spin-singlet excitation is converted into two overall spin-triplet excitations [2,6,7]. A critical intermediate in singlet fission is the correlated triplet pair. Because the correlated triplet pair is also overall spin singlet in nature, the formation of the correlated triplet pair can take place on the femtosecond and picosecond timescale [8,9,10,11,12,13,14,15,16,17,18], much faster than the timescale of intrinsic radiative and nonradiative decay of conjugated organic molecules. Thus, the correlated triplet pair, and subsequently two triplet excitations, [4,19,20,21,22,23] can be produced from one optical singlet excitation with high efficiency.

Perylene is a rigid polycyclic aromatic hydrocarbon with exemplary photophysical properties that make it an ideal candidate for singlet fission. For example, dilute solutions of perylene monomer in toluene and acetonitrile exhibit peak extinction coefficients of 39,000 and 37,000 M^−1^ cm^−1^, respectively, [24,25] in the visible wavelength region. Such appreciable peak extinction coefficients, which can correspond to appreciable transition dipole moment amplitudes, may be beneficial for forming delocalized, collective excitations, i.e., molecular excitons [26,27]. Additionally, in organic solvents such as toluene and acetonitrile, perylene exhibits a fluorescence quantum yield (FQY) of near unity, [24,28,29] indicating that perylene in its monomeric form exhibits very little intrinsic nonradiative decay. The minimal nonradiative decay in monomeric perylene is beneficial for singlet fission, as it minimizes parallel competing decay pathways. Furthermore, the energetics of perylene are such that it is capable of undergoing endothermic singlet fission. Specifically, singlet fission requires that the lowest-energy monomer triplet state be approximately one-half the energy of the lowest-energy singlet state, i.e., Δ*E*_SF_ ≈ 2 × *E*_T1_ − *E*_S1_^2^. In the case of unsubstituted perylene, *E*_S1_ = 2.83–2.86 eV (refs. [24,25,30]) and *E*_T1_ = 1.50 eV (ref. [31]). Thus, Δ*E*_SF_ ~ 0.1–0.2 eV. Therefore, unsubstituted perylene is capable of endothermic singlet fission, as has been shown in both α- and β-forms of the unsubstituted perylene crystal [31]. Moreover, its triplet energy of 1.5 eV is advantageous as the two triplet excitations resulting from singlet fission can potentially be transferred to an inorganic semiconductor, such as crystalline silicon, to generate two electron-hole pairs [32,33,34].

Key to singlet fission is the formation of molecular aggregates. There are a number of ways of inducing aggregation. Historically, spontaneous aggregation has been a convenient method of inducing aggregation [35,36]; however, it is limited in its ability to control the size of the aggregate and the molecular-level packing arrangements. Another method is to template the aggregation with a larger macromolecule. One such example is found in nature via proteins [37,38], which are central to molecular aggregation in photosynthetic light harvesting complexes [38]. However, the designed control of protein-templated molecular aggregation presents itself as a significant challenge, due the complexity of the protein folding when additional molecules that are not amino acids are included in the system. At present, a more tractable class of biomacromolecules are oligonucleotides, such as deoxyribonucleic acid (DNA). DNA consists of a small basis set of four nucleotides, all of which pair up via Watson–Crick base pairing. Even though the basis set is small, the possibilities for different structures are extensive [39,40,41,42,43,44]. For example, there are over 10^6^ different configurations for an oligonucleotide sequence consisting of just 10 nucleotides. Additionally, recent work has shown that controlling molecular aggregate packing on the nanoscale is possible in DNA-templated molecular aggregates [45,46,47,48,49,50,51,52,53,54,55,56,57,58,59,60,61,62,63,64,65,66,67].

In this work, we examine the electronic structure and excited-state dynamics of monomers and dimers of a perylene derivative templated using DNA. Specifically, DNA Holliday junctions (HJs) were used to template the formation of a perylene monomer, transverse dimer, and adjacent dimer. The electronic structure of the perylene monomer, transverse dimer, and adjacent dimer was characterized via steady-state absorption and fluorescence emission spectroscopy, which also provided insights into the packing arrangements in the two dimers. Relative fluorescence intensity measurements were also performed, which provided initial insight into the excited-state dynamics of the dimers compared with the monomer. The results indicated that a new nonradiative decay pathway is introduced in the dimers that is not present in the monomer. Time-correlated single photon counting (TCSPC) measurements were also performed and the results corroborated those of the relative fluorescence intensity measurements. Lastly, femtosecond visible (VIS) transient absorption (TA) was used to further characterize the excited-state dynamics of the monomer, transverse dimer, and adjacent dimer, and provide insight on the mechanism of the nonradiative decay introduced upon formation of the dimers. Based on key observations in the femtosecond VIS TA, the mechanism of nonradiative decay was proposed to result from symmetry-breaking charge transfer rather than singlet fission.

## 2. Materials and Methods

### 2.1. DNA-Dye Construction Preparation

NUPACK was employed to determine the mean free energy of the complementary base pairing of the oligonucleotide sequences used to assemble the DNA HJs [68]. Oligonucleotides labeled with dU-ethynyl-perylene (Glen Research, Sterling, VA, USA), which was incorporated into the oligonucleotide sequence via a modified uracil nucleobase, were purified by high-performance liquid chromatography and obtained as lyophilized powders from Integrated DNA Technologies (IDT; Coralville, IA, USA). Unlabeled oligonucleotide sequences were purified by standard desalting and were also obtained from IDT as lyophilized powders. Stock solutions of the labeled and unlabeled oligonucleotides were prepared at a concentration of ~100 μM by hydrating with water obtained from a Barnstead Nanopure water purification system (ThermoFisher Scientific, Waltham, MA, USA). The concentration of the stock solutions was determined according to Beer’s law by measuring the absorbance, using the extinction coefficient of the solution, and a scaled path length of 1 cm. The extinction coefficient at 260 nm was provided by the vendor and the absorbance of the solution was measured using a NanoDrop One UV-Vis spectrophotometer (ThermoFisher Scientific), which reports absorbance from a scaled path length of 1 cm. Solutions of DNA HJ templated monomer and dimers were prepared by combining equimolar amounts of the stock oligonucleotide solutions with a concentrated aqueous buffer solution consisting of 10× TAE and 150 mM MgCl_2_ and diluted with water for a final buffer concentration of 1× TAE and 15 mM MgCl_2_. After dilution, the final concentration of the DNA HJ templated monomer and dimers was ~4 μM. The resulting solutions were vortex mixed for 30 s and allowed to hybridize overnight in a dark container at room temperature.

### 2.2. Steady-State Absorption Spectroscopy

Steady-state absorption spectra were measured using a Cary 5000 UV-Vis spectrometer (Agilent Technologies, Santa Clara, CA, USA). Absorption measurements were collected from 230 to 650 nm in 1 nm steps averaging for 1 ms per step. The solutions were diluted for a maximum absorbance of 0.3 in the range of 350 to 500 nm.

### 2.3. Steady-State Fluorescence Spectroscopy

Steady-state fluorescence emission spectra were measured using a Fluorolog-3 spectrofluorometer (Horiba Scientific, Edison, NJ, USA). Solutions were excited at 450 nm with the entrance and exit slits set to a 1.5 nm bandpass. Fluorescence emission was collected from 470 to 700 nm in 1 nm steps with an averaging time of 1 ms per step. All fluorescence emission measurements were carried out in a 1 cm quartz cuvette (Starna Cells, Atascadero, CA, USA). For these measurements, a portion of the solutions initially prepared at 4 μM were diluted such that their maximum absorbance was 0.1 in the range of 350 to 500 nm.

### 2.4. Time-Correlated Single Photon Counting

Time-correlated single photon counting (TCSPC) measurements were conducted using a FluoTime 250 fluorescence spectrometer (PicoQuant, Berlin, Germany). The solutions were excited at 405 nm and emission was collected at ~500 nm, which corresponded to the peak emission intensity of the solutions. The instrument response function (IRF) was determined to be ~80 ps by measuring scattered light from a solution of colloidal silica (i.e., Ludox) located at the sample position. The sample solutions were diluted from their original concentration of 4 μM such that their maximum absorbance was 0.1 in the range of 350 to 500 nm.

### 2.5. Femtosecond Visible Transient Absorption Spectroscopy

A custom-built transient absorption (TA) spectrometer was used to measure all TA spectra. The initial stage of the TA light source is a Ti:sapphire-based fs laser oscillator (Coherent, Santa Clara, CA, USA), which produces an 80 MHz train of pulses centered at ~800 nm with a pulse energy of ~4 nJ/pulse. The output of the oscillator is used to seed a Ti:sapphire-based regenerative amplifier (Coherent), which produces a 1 kHz train of ~40 fs pulses centered at 800 nm that have been amplified to ~3 mJ/pulse. A portion of the regenerative amplifier output drives an optical parametric amplifier (Coherent), which was used to generate the 480 nm pump beam. A small fraction of the regenerative amplifier output was also used to generate the probe beam by focusing onto a 2 mm thick sapphire window (Newlight Photonics, Toronto, ON, Canada), producing a white light continuum spanning ~440 to 750 nm (Section S1). The pump and probe beams were spatially overlapped at the position of the sample. The relative time delay between the pump and probe at the sample position was controlled by varying the pump beam path length using a mechanical delay stage (Aerotech, Pittsburg, PA, USA). The TA spectra were collected by directing the probe beam into a spectrograph, which included a monochromator and a sCMOS array detector (Andor, Belfast, Northern Ireland). All measurements were carried out with the relative orientation of the linearly polarized pump and probe beams set to the magic angle of 55°. The beam power was measured in the presence and absence of a 50 μm diameter high-energy pinhole (Newport, Santa Clara, CA, USA) situated at the focal plane of the overlapped beams to estimate diameters of ca. 240 and 160 μm for the pump and probe beams, respectively. Pump pulse energies of ~0.5 µJ/pulse were used, which corresponded to a pump fluence of ~1.2 mJ/cm^2^. All solutions were contained in a 2 mm quartz cuvette (Starna Cells) and stirred with a magnetic stirrer bar (Starna Cells) using a magnetic stirring apparatus (Ultrafast Systems, Sarasota, FL, USA) over the course of the measurement. The pump pulse duration was ~190 fs for all measurements, as was determined by performing an autocorrelation of the pump with a 2 mm quartz cuvette filled with distilled water and situated at the sample position (Section S1).

Measurements extending into the several nanosecond timescale were obtained on a TA spectrometer that has been described in detail previously [64,65].

## 3. Results and Discussion

**Scheme 1:**
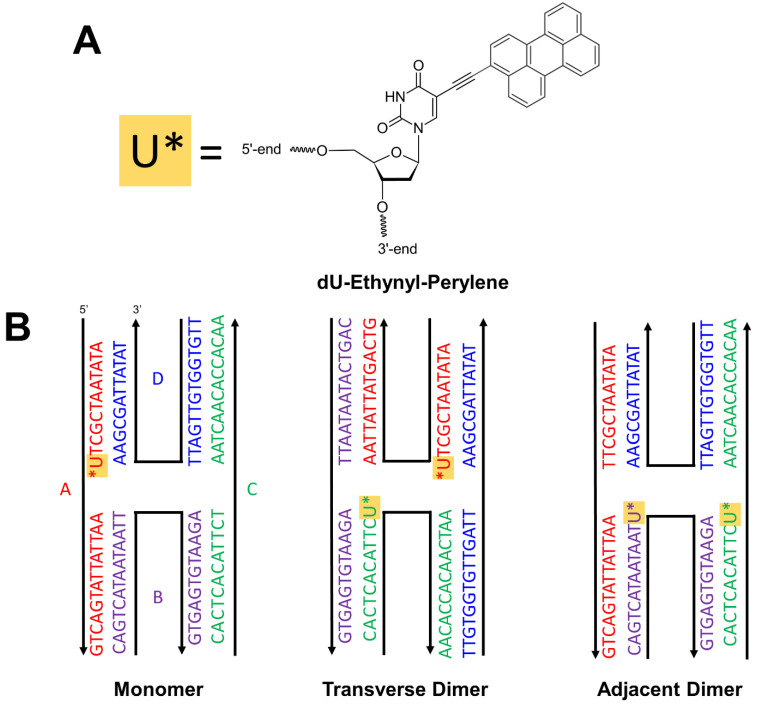
(**A**) The conjugated organic molecule perylene, which can be incorporated into an oligonucleotide sequence via covalent attachment to a uracil nucleobase through an ethynyl substituent located on the short axis of the molecule (i.e., dU-Ethynyl-Perylene [U*]). (**B**) Schematic representation of the “immobile” four-armed DNA HJs [39] used to template the formation of perylene monomer, transverse dimer, and adjacent dimer. The DNA HJs are composed of four oligonucleotide sequences color-coded and labeled A (red), B (purple), C (green), and D (blue). Each sequence is reported next to a black arrow, where the 5′ end is located at the end of the arrow and the 3′ end is located at the head of the arrow. The lettering scheme corresponds to the nucleobases: adenine (A), cytosine (C), guanine (G), thymine (T), and uracil (U). The integration of U* (described in panel A) in the oligonucleotide sequence is highlighted with a gold box. The four-armed DNA HJs can preferentially take on one of two conformations, open or stacked, depending on the solution conditions. Here, the DNA HJs are depicted in a stacked conformation given the similarity of the oligonucleotide sequences and solution conditions to that of prior work [58], which showed that the DNA HJs preferentially take on a stacked conformation in solutions containing the divalent cation Mg^2+^.

Perylene was chosen as an exemplary chromophore potentially capable of singlet fission that can be covalently attached to DNA (Scheme 1A). In DNA templating, typical linkers include dual phosphoramidite linkers [49,50,54,58,60,65], where the chromophore can be inserted as part of the oligonucleotide sequence, or a single NHS ester linker [61,63,64], where the chromophore is covalently linked to a nucleobase that is inserted as part of the oligonucleotide sequence. Here, perylene is covalently bound to the oligonucleotide via an ethynyl bond that directly connects a uracil nucleobase to a carbon atom on the short axis, i.e., the peri-side, of the molecule. Perylene was templated in the form of a monomer and two different types of dimers, i.e., transverse and adjacent, via four-armed DNA Holliday junctions (Scheme 1B). A specific oligonucleotide sequence was designed to ensure complementary pairing between nucleobases, including between uracil and adenine, as can be seen in the primary sequences displayed in Scheme 1B.

**Figure 1:**
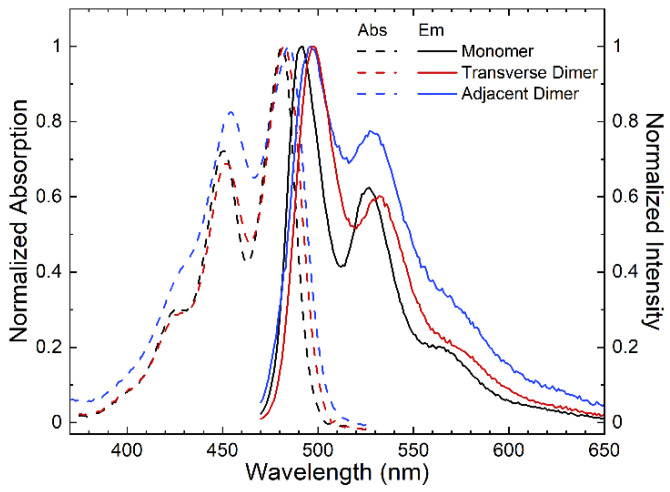
Steady-state absorption and fluorescence emission spectra of DNA-templated perylene monomer (black), transverse dimer (red) and adjacent dimer (blue) solutions. The absorption and fluorescence emission spectra are plotted with dashed and solid lines, respectively, and are normalized to their corresponding most intense absorption and fluorescence emission bands.

To characterize the photophysical behavior of the DNA-templated monomer and dimers, we first performed steady-state absorption and fluorescence spectroscopy (Figure 1). The DNA-templated perylene monomer features its most-intense absorption band at ~480 nm, which is redshifted with respect to that of unsubstituted perylene monomer in solution. For example, the peak absorption wavelengths of unsubstituted perylene in the nonpolar solvent toluene is ~438 nm (see, e.g., refs. [24,30]) and in the polar solvent acetonitrile is ~434 nm (ref. [25]). In alignment with prior work [69], the redshifted absorption profile of the DNA-templated perylene monomer is attributed to extended π-conjugation through the ethynyl linker and uracil nucleobase used to attach the chromophore to the oligonucleotide sequence (Scheme 1A). The absorption spectrum exhibits additional, less-intense absorption bands at shorter wavelengths of ~450, 430, and 390 nm. The energy difference between these absorption bands is appx. 1700 cm^−1^, which is assigned to coupling of the electronic transition to a carbon-carbon stretching mode. The absorption bands at ~480, 450, 430, and 390 nm are assigned to 0–0, 0–1, 0–2, and 0–3 vibronic bands, respectively, associated with this mode. The maximum extinction coefficient for the DNA HJ templated perylene monomer is ~63,000 M^−1^ cm^−1^, which corresponds to a derived transition dipole moment amplitude of ~7 D (Section S2). Additionally, the DNA-templated perylene monomer exhibits its most-intense fluorescence emission band at ~490 nm and a fluorescence emission spectrum that largely mirrors the absorption profile. Taking the energy difference between the lowest-energy absorption and highest-energy fluorescence emission bands (that correspond to the most-intense absorption and fluorescence emission bands), a value of ~420 cm^−1^ is obtained for the Stokes shift. Literature reports a much smaller Stokes shift of 260 cm^−1^ for perylene in both acetonitrile and toluene [24,25,29]. It is possible that the larger Stokes shift observed in the DNA-templated perylene arises from: (i) a break in the symmetry of perylene with the addition of the ethynyl linker and uracil nucleobase, or (ii) the incorporation of perylene into the DNA HJ nanostructure [69,70].

The steady-state absorption and fluorescence emission spectra of the DNA-templated perylene transverse and adjacent dimer solutions are also displayed in Figure 1. The dimer solution spectra are qualitatively similar to the monomer spectra, and the fluorescence emission spectra of the dimers also largely mirror their corresponding absorption spectra. Both absorption and fluorescence emission spectra of the dimers are redshifted compared with the corresponding spectra of the monomer. Perhaps the most notable differences in the monomer and dimer absorption and emission spectra are related to variations in their vibronic absorption band amplitudes. Specifically, when compared with the monomer, the amplitude of the 0–1 absorption and fluorescence emission bands is smaller and larger for the transverse and adjacent dimer, respectively. Table 1 displays *A*_0–0_/*A*_0–1_ and *I*_0–0_/*I*_0–1_ ratios for the monomer, transverse dimer, and adjacent dimer solutions. Clearly, the *A*_0–0_/*A*_0–1_ and *I*_0–0_/*I*_0–1_ ratios increase and decrease for the transverse and adjacent dimer, respectively. These observations, particularly the variations in the relative vibronic absorption and fluorescence emission intensity, are consistent with weak excitonic interactions between perylenes within the dimer [71,72,73,74]. Furthermore, current models of exciton theory predict that an increase (decrease) in *A*_0–0_/*A*_0–1_ and *I*_0–0_/*I*_0–1_ ratios corresponds to J- (H-) aggregate packing. The results indicate that the perylene molecules in the transverse and adjacent dimers exhibit weak excitonic interactions and exhibit J- and H-aggregate packing, respectively.

**Figure 2:**
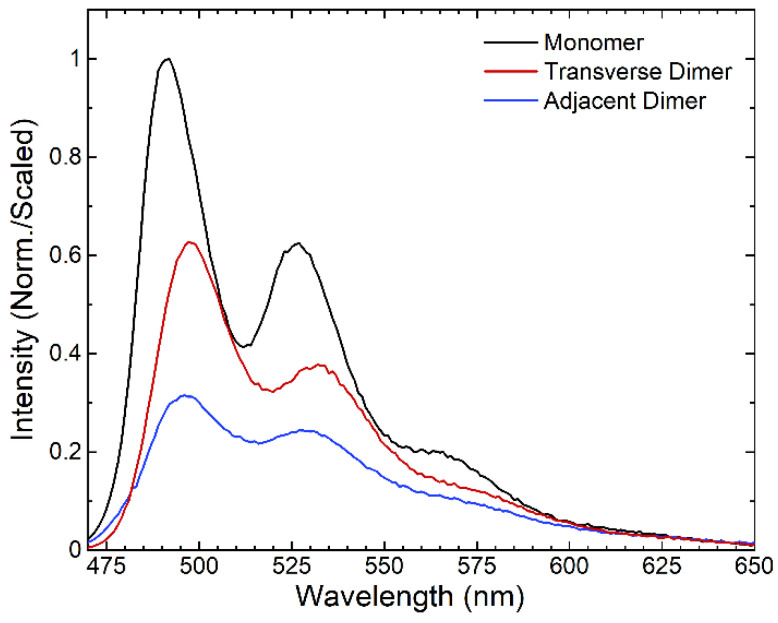
Relative fluorescence emission spectra of DNA-templated perylene monomer, transverse dimer, and adjacent dimer solutions. The fluorescence emission spectra were all scaled by dividing by the absorptance of the solution at the excitation wavelength (see, e.g., Experimental Methods). The monomer spectrum was subsequently normalized to the most intense emission feature. The transverse and adjacent dimer spectra were further scaled using the normalization factor determined for the monomer.

To investigate if any nonradiative decay pathways, such as singlet fission, are introduced upon forming the perylene dimers, we performed relative fluorescence intensity measurements. Figure 2 shows that the monomer solution exhibits the most intense fluorescence emission, followed by the transverse dimer and then the adjacent dimer solutions, i.e., *I*_monomer_ > *I*_transverse dimer_ > *I*_adjacent dimer_. The observed trend in the fluorescence emission intensities indicates that a new nonradiative decay pathway is introduced in the perylene dimers, with the nonradiative decay pathway being more significant in the adjacent dimers.

**Figure 3:**
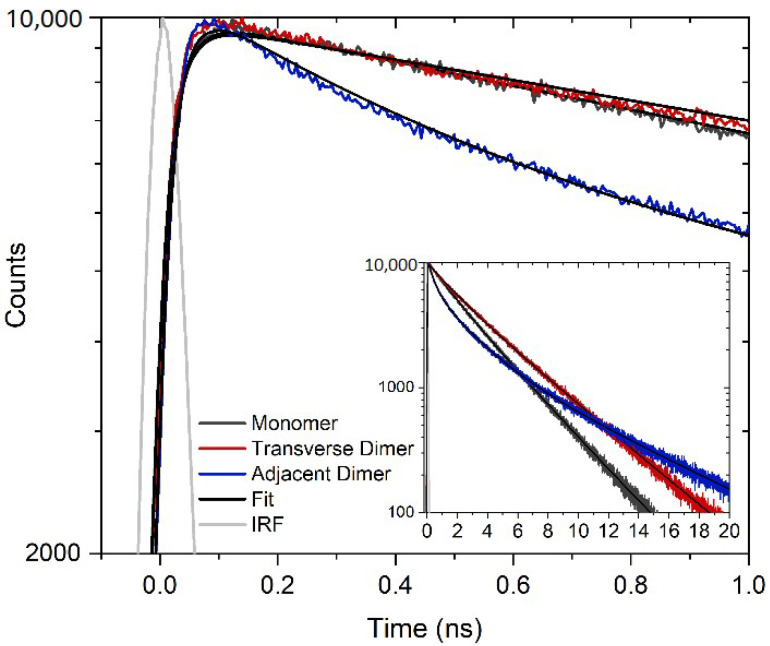
TCSPC decay traces obtained for DNA HJ templated perylene monomer, transverse dimer, and adjacent dimer solutions. The solutions were excited at 405 nm and fluorescence emission was collected at 497 nm. A zoomed-out plot of the same data is shown in the inset.

Time-resolved fluorescence spectroscopy was used to further characterize the nonradiative decay concomitant with aggregation of perylene. Figure 3 displays TCSPC measurements of the DNA-templated monomer, transverse dimer, and adjacent dimer solutions. The solutions were excited at 405 nm and fluorescence emission was detected at ~500 nm. The monomer fluorescence emission decay trace was best described by a two-exponential fit, which yielded time constants of 1.69 and 3.39 ns and associated amplitudes of 0.26 and 0.74, respectively. The amplitude-weighted average of the time constants was 2.9 ns, which is attributed to the excited-state lifetime of perylene monomer templated to DNA. In bulk acetonitrile and toluene, the fluorescence decay is reported as a single exponential decay with a lifetime of 4.2 and 3.9 ns, respectively [24,29,69]. However, once the ethynyl linker is attached to the perylene molecule, a biexponential decay was observed with fluorescence lifetimes of 1.2 and 3.3 ns with associated amplitudes 0.39 and 0.61, respectively [69]. When a weighted average is applied, the two lifetimes were reduced to 2.5 ns, which is similar to the present results. In the case of the transverse and adjacent dimers, the fluorescence emission decay traces were best described by a three-exponential fit. In both cases, a short sub-nanosecond time constant was derived, along with two longer nanosecond timescale time constants (Table 2). The amplitude associated with the short sub-nanosecond time constant was ~0.12 and 0.39 for the transverse and adjacent dimer, respectively. The presence of a short sub-nanosecond time constant indicates that an effective nonradiative decay pathway is present in both transverse and adjacent dimer solutions, and the increase in amplitude in the adjacent dimer solution is consistent with the nonradiative decay playing a larger role in the photophysics of the adjacent dimer. Additionally, taking the amplitude-weighted average of the latter two time constants results in values of ~3.8 and 3.9 ns for the long-lifetime components of the fluorescence emission decay traces of the transverse and adjacent dimer solutions, respectively. These time constants are assigned to a subpopulation of perylene monomers present in the transverse and adjacent dimer solutions, given that their amplitude-weighted average values are generally consistent with the lifetime of the perylene monomer [24,29,69].

**Figure 4:**
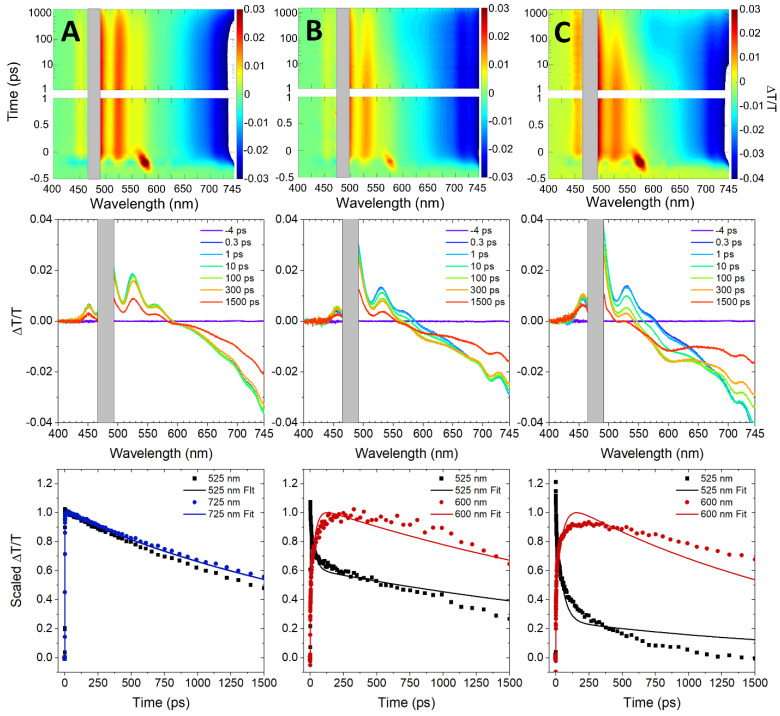
Femtosecond visible transient absorption of DNA-templated perylene monomer, transverse dimer, and adjacent dimer solutions excited at 480 nm. The vertical columns correspond to (**A**) monomer, (**B**) transverse dimer, and (**C**) adjacent dimer solutions. Surface plots are on top, selected spectra in the middle, and selected kinetics shown on the bottom. The gray box in the surface plots and selected spectra overlays regions of the spectra contaminated by dynamic pump scatter. The selected kinetics plots correspond to stimulated emission (525 nm) and excited-state absorption features (600 nm and 725 nm) with corresponding fits from global target analysis overlaying the kinetics traces.

Femtosecond visible (VIS) transient absorption (TA) was used to further investigate the mechanism of nonradiative decay observed in the perylene transverse and adjacent dimer solutions. Figure 4 displays the femtosecond VIS TA of the perylene monomer, transverse dimer, and adjacent dimer solutions. The TA spectra are plotted as change in transmittance, i.e., ∆*T*/*T*. In the case of the monomer, prominent positive-amplitude signals are observed in the selected transient spectra at 525 and 565 nm, which are assigned to stimulated emission (SE) features. Additionally, intense negative-amplitude signals are observed above ~600 nm, which are assigned to excited-state absorption (ESA) features. The observation of intense ESA features above ~600 nm for the DNA-templated perylene monomer is consistent with Furube et al. who observed intense ESA features above ~560 nm in dilute solutions of unsubstituted perylene in toluene [75]. Due to limitations of the spectral range of the VIS probe continuum as well as dynamic pump scatter, positive-amplitude ground-state bleach (GSB) features were only observed weakly at ~450 nm. A fit from a global target analysis (GTA) of the monomer femtosecond VIS TA overlays the selected transient kinetics shown in Figure 4A. GTA is a form of analysis where: (i) the data are analyzed globally, i.e., the kinetics at all wavelengths are analyzed simultaneously, and (ii) a single model is chosen that is determined to be the best mathematically suited to model the data and most physically appropriate to describe the excited-state dynamics of the system [76,77]. As shown in Table 3, the analysis derives a lifetime >1.5 ns, i.e., the duration of the measurement, which is consistent with the ~2.9 ns lifetime measured via TCSPC (Table 2).

At the earliest measured timescales (i.e., ~300 fs), the TA spectra of the perylene transverse and adjacent dimer solutions exhibit signals that are overall largely similar to the TA spectra of the monomer. For example, SE features are observed at 525 and 565 nm, ESA features are observed above ~580 nm, and a GSB feature is observed at 450 nm. At longer timescales, the TA spectra of perylene transverse and adjacent dimers clearly evolve in a distinct manner. Specifically, a new negative-going signal at ~600 nm grows in. Based on its sign, the signal is assigned to an ESA feature. Interestingly, the amplitude associated with this feature is more significant in the case of the adjacent dimer relative to the transverse dimer. These observations are most evident in the selected kinetics traces at 525 and 600 nm plotted in the bottom row of Figure 4. Additionally, Figure 4 shows that the trace at 525 nm (that corresponds to the SE feature of the perylene dimers) decays rapidly on the 10 s of ps timescale and, concomitantly, the trace at 600 nm (that corresponds to the ESA feature of the perylene dimers) grows in rapidly on the same timescale. These results are a direct confirmation of a new nonradiative decay that emerges in the perylene dimer aggregates (i.e., that are not present for the monomer) and provide a potential clue as to its origin via the observation of a new ESA feature peaking at ~600 nm.

A GTA of the femtosecond VIS TA was undertaken to provide additional details on the excited-state dynamics of the transverse and adjacent dimer solutions. Table 3 shows that the GTA of the adjacent and transverse dimers were suitably described by two time constants: (i) a small time constant of ~30 and 50 ps for the transverse and adjacent dimer solutions, respectively, and (ii) a large time constant of >1.5 ns for both solutions (see Section S3 for additional details on the GTA). As seen in Figure 4, the kinetics traces at 525 and 600 nm for both dimer solutions exhibit rapid change at early time delays. The first time constant is assigned to the initial perylene dimer excitation that transitions to a non-emissive state on a ~30–50 ps timescale. The imperfect match of the GTA fit with the data may be because there is a distribution of time constants needed to describe the dynamics, which may originate from a distribution of orientations between dyes in the weakly excitonically coupled, DNA-templated dimers. Such nonexponential excited-state dynamics have been previously observed in weakly excitonically coupled systems, i.e., in amorphous solids [11,21] and micelles [78] of pentacene derivatives and in propyl-linked perylene dimers [79]. In the case of the propyl-linked perylene dimers, the nonexponential excited-state dynamics were attributed to a distribution of conformations arising from the conformational flexibility of the dimers. In the case of the DNA-templated perylene dimers, a distribution of orientations may originate from a distribution of packing angles and/or distances, the latter of which was proposed to explain variations in the excited-state lifetime of DNA-templated cyanine aggregates [60]. For simplicity, the first (single) time constant derived by the model is taken to be an adequate representation of the effective or average timescale of the initial excited-state dynamics of the dimers (Section S3). The second time constant in the model is assigned to a combination of the lifetime of the resultant non-emissive state along with that of a subpopulation of perylene monomers excited in parallel with the perylene dimers. The latter assignment is based on the following: (i) the lifetime of the non-emissive state, i.e., see more on the assignment below, is assumed to be longer than the duration of the measurement, (ii) the >1.5 ns time constant is consistent with the excited-state lifetime of the perylene monomers observed in TCSPC, and (iii) the long-time transient spectra of the perylene dimer solutions exhibit considerable amplitude in the vicinity of the SE features associated with the perylene monomers, i.e., ~500 and 525 nm, along with considerable amplitude in the vicinity of their ESA features, i.e., ~710 and 745 nm. Similar observations of monomer photophysics taking place in parallel with dimer photophysics have been observed previously in solutions of DNA-templated cyanine, squaraine, and squaraine rotaxane aggregates [54,60,61,64].

**Figure 5:**
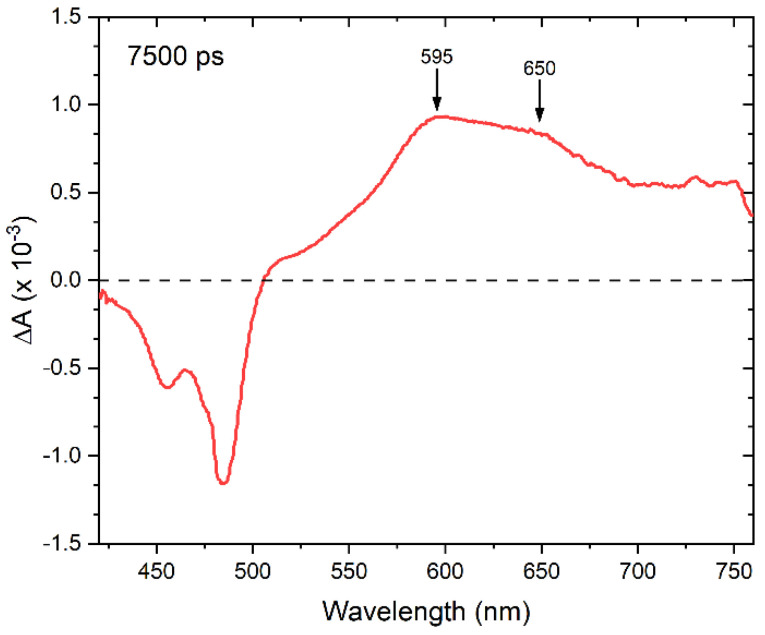
Femtosecond visible transient absorption spectrum of a solution of DNA-templated adjacent dimer obtained at ~7.5 ns.

There are several possible assignments for the new ESA feature that appears in the perylene dimer solutions at ~600 nm on the ~30–50 ps timescale. To gain additional insight, VIS TA measurements extending out to the several nanosecond timescale were undertaken. Figure 5 displays the VIS TA spectrum acquired at ~7.5 ns for the DNA-templated adjacent perylene dimer solution and represented as Δ*A*, which is equivalent to −Δ*T*/*T* in the limit that the pump-induced change in signal intensity is small compared with the overall signal intensity [80]. From a practical perspective, the sign of the various features contributing to the TA signal are inverted, i.e., GSB and SE features become negative signals and ESA features become positive signals. On the 7.5 ns timescale, the parallel-decaying monomer signal has decayed considerably (i.e., ~15% or less of the signal remains), providing a largely unobstructed view of the spectral profile of the species resulting from the nonradiative decay. The spectrum at 7.5 ns exhibits GSB and ESA features, while SE features are clearly absent, which is consistent with the resultant species being non-emissive. Critically, the ESA features above ~510 nm are broad and largely featureless, with the most intense absorption appearing at ~595 nm and a secondary maximum appearing at ~650 nm.

The observation of two ESA features in the 7.5 ns VIS TA spectrum is consistent with the formation of anions and cations in the DNA-templated perylene dimers. For example, Kawai et al. [81] and others [82,83,84] have shown that that the triplet ESA (i.e., *T*_1_ → *T*_n_) associated with unsubstituted perylene exhibits a peak maximum at ~490 nm, with additional, less intense vibronic structure at shorter wavelengths (i.e., similar to *S*_0_ → *S*_1_; see, e.g., Figure 1). Even if the triplet ESA were redshifted owing to the extended conjugation of dU-Ethynyl-Perylene, it would not explain the breadth of the spectral features displayed in Figure 5 along with their relative intensity, where the band peaking at ~595 nm band is more intense than the band peaking at ~650 nm. Specifically, in the case of the perylene triplet ESA the most intense feature is expected to be the longest wavelength, with the shorter wavelength features exhibiting less intensity [81]. Thus, these observations are not consistent with the assignment of the ESA features to triplet excitations. Secondly, Cook et al. examined the excited-state dynamics of a series of perylene dimers covalently linked via a xanthene bridge [85]. The authors showed that excimer relaxation was the primary decay pathway and that the resultant excited state exhibited a single, featureless ESA band peaking at ~620 nm. While the process of excimer relaxation is expected to be nonradiative, the resultant “excimer” can exhibit efficient radiative coupling to the ground state, i.e., be highly emissive [86]. As such, a characteristic signature of excimer relaxation is the presence of a largely featureless, broadened, and appreciably redshifted band (compared with the corresponding monomer) in the steady-state fluorescence emission spectrum [85,87]. These observations are in contrast to those reported here, and so the assignment of the ESA features to perylene “excimers” is also ruled out. Lastly, anions and cations of unsubstituted perylene are known to absorb at ~595 and 540 nm, respectively [79,81,88]. These species each exhibit a single, intense and featureless *D*_0_ → *D*_5_ band, whose TDM is oriented along the long axis of perylene. If the TDM amplitude associated with the absorption is assumed to be similar for the *D*_0_ → *D*_5_ absorption of the anionic and cationic forms of perylene, similar redshifts may be expected for each transition for the extended conjugation in dU-Ethynyl-Perylene. Taking the peak maxima of the cationic and anionic forms of unsubstituted and substituted perylene to be ~540 and 580 nm and 595 and 650 nm, respectively, nearly equivalent redshifts of ~1710 cm^−1^ are obtained, which is consistent with this interpretation. Thus, the two ESA features appearing in the 7.5 ns VIS TA spectrum are assigned to perylene anions and cations, likely resulting from charge transfer (CT) between perylenes in the DNA-templated dimer.

That CT would explain the nonradiative decay in the DNA-templated perylene dimers is additionally supported by considering the kinetics associated with the formation of the new ESA features. As noted above, one form of nonradiative decay that is possible in perylene dimers is the formation of triplet excitations due to singlet fission. Singlet fission in dimers of unsubstituted perylene is expected to be endothermic with an energy barrier of 0.1–0.2 eV (i.e., Δ*E_SF_* = 2 × *E_T_*_1_ − *E_S_*_1_) because the energy of the lowest-singlet state of unsubstituted perylene is ~2.83–2.87 eV and the lowest-triplet state energy is ~1.5 eV. For example, Korovina et al. synthesized and characterized a series of phenylalkynyl-linked, linear perylene oligomers and observed endothermic singlet fission [89]. Because of the endoergic energy barrier, the triplet excitations that formed in the linear perylene trimers and tetramers exhibited formation time constants of ~2–3 ns. The long formation time constants associated with triplet excitations resulting from singlet fission in these perylene trimer and tetramer aggregates are not in agreement with the ~30–50 ps time constants for the DNA-templated perylene dimers. Therefore, the formation of triplet states via singlet fission appears unlikely to explain the nonradiative decay observed in the DNA-templated perylene dimers.

Alternatively, the nonradiative decay may be attributed to CT, which can take place on a more rapid, picosecond timescale. For example, CT can occur in molecular dimers through a process called symmetry breaking (SB) CT [79,90,91]. In SB CT, the environment induces local electric fields at either or both ends of the dimer that stabilize the energy of anions and cations and facilitate their formation. As one example, propyl-tethered perylene dimers dissolved in acetonitrile have been observed to form anions and cations on the tens of picoseconds timescale via SB CT [79]. In this system, SB CT was explained by considering that the average solvent response time of acetonitrile is <250 fs [90], which, via random fluctuations, may permit solvent configurations that stabilize the formation of anions and cations on the two perylenes over the lifetime of the electronic excited state. The authors of the work further used polarization-resolved VIS TA to show that CT was bidirectional, i.e., both photoinduced electron and hole transfer took place with equal probability. On the other hand, SB CT was originally invoked to explain the unidirectional nature of CT in the reaction center of photosynthetic bacteria [90,92,93]. Specifically, light energy is funneled to a bacteriochlorophyll (BChl) dimer, known as the special pair, where CT can proceed along one of two branches with equivalent electron-accepting BChl monomers. However, CT takes place only along one of these branches, with the initial step occurring on a few picosecond timescale. These observations have been rationalized by considering that the protein environment in one of the branches may be configured in a manner to stabilize the formation of anions and cations on the electron acceptor and donor, respectively. Therefore, CT is unidirectional, i.e., only electron transfer takes place. Similarly, Tsuboi et al. have shown that SB CT takes place on a few picosecond timescale in 9,9′-bianthryl adsorbed on porous glass [94]. One way to explain the results is to consider that at the 9,9′-bianthryl:glass interface, there may be fixed local electric fields conducive to SB CT. Such a scenario may also permit unidirectional CT. In the case of the DNA-templated perylene dimers at the focus of the present work, SB CT may either be bidirectional due the presence of a highly polar aqueous buffer solution or it may be unidirectional due to the covalent attachment of the perylenes to the DNA template. While it is beyond the scope of the current work to determine whether CT is bidirectional or unidirectional in the DNA-templated perylene dimers, clearly the formation of anions and cations can readily be explained by SB CT.

Having assigned the origin of the nonradiative decay in the dimers to SB CT, the results are next rationalized and the implications are discussed. As previously noted, singlet fission has been observed in both α- and β-forms of the unsubstituted perylene crystal (ref. [31]) and in phenylalkynyl-linked perylene trimers and tetramers [89], which motivated the present study. There are several reasons that may explain why SB CT was observed in the DNA-templated perylene dimers rather than singlet fission. In a seminal study examining the interplay of singlet fission, excimer relaxation, and SB CT, Margulies et al. synthesized a series of triptycene bridged terrylenediimide dimers with varying slip-stacking distances [15]. In the most strongly coupled dimers, which exhibited the smallest slip-stacking distances and most drastic changes to their absorption spectra, primarily excimer relaxation was observed on a ≤200 fs timescale. In contrast, in the weakly coupled dimer, which exhibited the largest slip-stacking distance (of ~7.6 Å) and modest changes to its absorption spectrum, singlet fission took place in the nonpolar solvent toluene (ε = 2.4) rather than excimer relaxation, while the formation of anions and cations via SB CT was favored in the polar solvent dichloromethane (ε = 8.9). The authors proposed that SB CT was observed in polar solvents rather than singlet fission because the energy of the solvated anions and cations was reduced such that it was lower than that of the correlated triplet pair. Thus, the formation of solvated anions and cations was favored over triplet pair formation. The DNA-templated perylene dimers at the focus of the present study were dissolved in a highly polar aqueous buffer (i.e., ε ~ 80), which may explain why the formation of anions and cations was preferred. Furthermore, much like the weakly coupled terrylenediimide dimers, the DNA-templated perylene dimers exhibited weak excitonic interactions (Figure 1), which may explain why excimer relaxation was not observed. In further support of this interpretation, Cook et al. synthesized a series of xanthene-bridged perylene dimers [85], which exhibited strong excitonic interactions, and observed that a primary decay pathway of the dimers dissolved in acetonitrile (ε = 38) was excimer relaxation. In contrast, Markovic et al. synthesized propyl-linked perylene dimers, which exhibited weak excitonic interactions, and showed that SB CT, rather than excimer relaxation, was the primary decay pathway in the same highly polar solvent [79]. Similar relationships between excitonic coupling, π-orbital overlap, excimer relaxation, and SB CT have been reported by Wu et al. in a covalently linked, triangular perylenediimide trimer aggregate [95]. Another reason that may explain why singlet fission was not observed in the DNA-templated perylene dimers is that higher-order perylene aggregates may be required to enable overall favorable energetics for singlet fission. For example, Korovina et al. showed that singlet fission took place in phenylalkynyl-linked perylene trimers and tetramers (dissolved in tetrahydrofuran, ε = 7.6), but did not take place in dimers [89]. The authors reasoned that the entropic contribution of the additional molecules in the trimer and tetramer favored the overall energetics of singlet fission, such that singlet fission was able to take place on the nanosecond timescale. Thus, it is possible that more molecules are needed in the DNA-templated perylene aggregate assembly to support singlet fission. Fortunately, the observation of SB CT in DNA-templated perylene dimers sheds promising light on the potential for singlet fission in these material systems, as it indicates that the perylenes are close enough to interact and—given the right system and environment conditions—might interact in a manner to undergo singlet fission. Such conditions may include synthesizing higher-order aggregates such as trimers and tetramers, which have been shown to be accessible via DNA templating methods [49,50,51,57,58,62,63], and DNA templating in the solid state [96], which should reduce the overall polarity of the environment.

## 4. Conclusions

In conclusion, DNA-templated perylene transverse and adjacent dimer aggregates were found to undergo symmetry-breaking charge transfer. A DNA-tethered perylene monomer, whose electronic structure and excited-state dynamics were characterized, was used as a control. The DNA-tethered perylene monomer exhibited steady-state and time-resolved behavior typical of a highly fluorescent dye, including intense fluorescence emission and long excited-state lifetime. In contrast, the DNA-templated perylene transverse and adjacent dimer aggregates exhibited successively less fluorescence emission intensity. These results were consistent with TCSPC measurements, which indicated a short, sub-nanosecond time constant present in both transverse and adjacent dimer solutions, with increasing amplitude in the adjacent dimer solution. Lastly, femtosecond VIS TA measurements identified an ESA band that peaks at ~600 nm and grows in on a ~30–50 ps timescale. Given the location of the spectral feature, along with the rapid timescale of its formation, the ESA band was attributed to anion and cation absorption resulting from symmetry-breaking charge transfer.

## Figures and Tables

**Table 1:** Steady-State Absorption and Fluorescence Emission Characterization of DNA-Templated Perylene Monomer and Adjacent and Transverse Dimers.

Structure	λ_abs, 0–0_ (nm)	λ_em, 0–0_ (nm)	Stokes Shift (cm^−1^)	Stokes Shift (meV)	*A*_0–0_ */A*_0–1_	*I*_0–0_ */I*_0–1_
Monomer	481	491	420	50	1.38	1.60
Transverse Dimer	482	496	590	70	1.45	1.67
Adjacent Dimer	485	495	420	50	1.21	1.29

**Table 2:** Time Constants and Amplitudes Derived from TCSPC Measurements of DNA-Templated Perylene Monomer, Transverse Dimer, and Adjacent Dimer Solutions.

Structure	τ_1_ (ns)	A_1_ (%)	τ_2_ (ns)	A_2_ (%)	τ_3_ (ns)	A_3_ (%)	τ_avg__2,3_ (ns)
Monomer	-	-	1.69	26	3.39	74	2.95
Transverse Dimer	0.28	12	2.50	31	4.49	57	3.79
Adjacent Dimer	0.39	39	2.21	40	7.25	21	3.89

**Table 3:** Time Constants Derived from a Global Target Analysis of Femtosecond Visible Transient Absorption of DNA-Templated Perylene Monomer, Transverse Dimer, and Adjacent Dimer Solutions.

Structure	τ_1_ (ps)	τ_2_ (ps)
Monomer	-	>1500
Transverse Dimer	30	>1500
Adjacent Dimer	50	>1500

## Data Availability

All data are available from the corresponding author upon request.

## Supplementary Materials

The following supporting information can be downloaded at: https://www.mdpi.com/article/10.3390/molecules27196612/s1. Section S1 contains additional details of the transient absorption spectrometer, including a spectrum of the white light continuum used as the probe beam as well as an autocorrelation of the pump beam. Section S2 describes how the transition dipole moment was calculated from the extinction spectrum. Section S3 describes the analysis of the transient absorption, which included global target analysis and manual biexponential fitting. Section S4 includes additional details of transient absorption measurements extending into the several nanosecond timescale.

## Sample Availability

Samples of the compounds are not available from the authors.
