# Supplementary material for: Symmetry Breaking Charge Transfer in DNA-Templated Perylene Dimer Aggregates
Source: Molecules. 2022 Oct 5;27(19):6612. doi: 10.3390/molecules27196612 (PMC9571668; doi:10.3390/molecules27196612)
Supplement: Supplementary file 1 [file molecules-27-06612-s001.zip › molecules-1924638-supplementary.pdf]

Supporting Information

Symmetry Breaking Charge Transfer in DNA-Templated Perylene Dimer Aggregates

Katelyn M. Duncan,[1] Donald L. Kellis,[1] Jonathan S. Huff,[1] Matthew S. Barclay,[1] Jeunghoon Lee,[1,2] Daniel B. Turner,[1] Paul H. Davis,[1,3] Bernard Yurke,[1,4] William B. Knowlton,[1,4] Ryan D. Pensack,[1]

[1]Micron School of Materials Science & Engineering, [2]Department of Chemistry & Biochemistry, and [4]Department of Electrical & Computer Engineering, Boise State University, Boise, Idaho 83725, United States

[3]Center for Advanced Energy Studies, Idaho Falls, ID 83401, United States

Table of Contents:

Section S1. Details of Femtosecond Visible Transient Absorption Spectroscopy

Section S2. Derivation of Perylene Monomer Transition Dipole Moment Amplitude

Section S3. Analysis of Visible Transient Absorption of Perylene Monomer, Transverse Dimer, and Adjacent Dimer Solutions

Section S4. Nanosecond Visible Transient Absorption of Adjacent Perylene Dimer Solution

Section S1. Details of Femtosecond Visible Transient Absorption Spectroscopy

For femtosecond visible (VIS) transient absorption (TA) spectroscopy, a white light continuum (WLC) was used as the probe. By focusing a small fraction of the regenerative amplifier output on a sapphire window, a WLC was generated. **Figure S1** is a typical WLC spectrum where any wavelengths longer than 800 nm were blocked by a 750 nm short pass filter to isolate the WLC from the fundamental output of the regenerative amplifier.

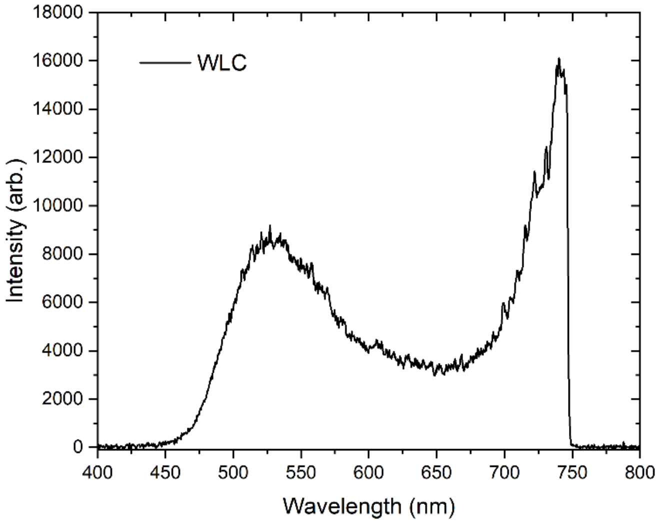

Figure S1. A typical spectrum of white light continuum probe used in transient absorption measurements.

Polarization gating was used to measure the pump pulse duration at the sample position (see e.g. Trebino *et al. Ref. Sci. Instrum.* **1997**, 68, 3277). The pump pulse duration was estimated by fitting the autocorrelation that resulted from the polarization gating measurement with a Gaussian function and calculating the full width at half maximum of the Gaussian function (**Figure S2**). The duration of the pump pulse was measured to be ~190 fs.

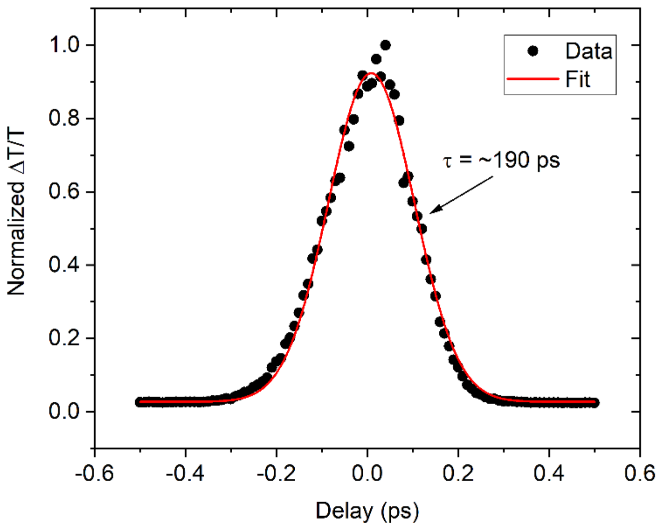

Figure S2. The autocorrelation resulting from polarization gating measurement of the 480 nm pump pulse duration, overlaid with a Gaussian function used to model the data and derive a pulse duration of ~190 fs.

Section S2. Derivation of Perylene Monomer Transition Dipole Moment Amplitude

Literature has reported the peak molar extinction coefficient for unsubstituted perylene to be 37,000 and 39,000 M^{-1} cm^{-1} in acetonitrile and toluene, respectively (see e.g. Hu *et al. J. Phys. Chem. B* **2021**, 125, 4187 and Carrod *et al. J. Mater. Chem. C* **2022**, 10, 4923). Linking perylene to ethynylene and a uracil base changes the peak molar extinction coefficient to 63,000 M^{-1} cm^{-1} in an aqueous 1× TAE buffer with 15 mM $MgCl_2$. While the peak molar extinction coefficient is a useful experimentally accessible property, a more generalized, microscopic property describing the ability for a chromophore to absorb light is the transition dipole moment (TDM). The TDM was calculated for uracil-ethynylene-linked perylene molecules used in this study according to **Equation S1** (see e.g. Chung *et al. Methods Appl. Fluoresc.* **2016**, 4, 045001 and Lewis *et al. Chem. Phys. Lett.* **1998**, 282, 197),

$$\text{TDM} = 9.58\,\text{x}10^{-2}\left(\frac{(2n^2+1)^2}{9n^3}\int\frac{\varepsilon(\nu)}{\nu}d\nu\right)^{1/2} \quad \textbf{(S1)}$$

where n is the refraction index for water (1.3327), ε is the molar extinction coefficient, and v is in units of wavenumber (cm^{-1}). The integral portion can be calculated by taking the area under the curve of the extinction spectrum plotted as a function of wavenumber (**Figure S3**). The extinction spectrum was calculated according to Beer's law, i.e., the absorbance at each wavelength was divided by the path length and concentration. **Equation S1** gives a TDM value in units of Debye (D). The TDM for the DNA-templated perylene monomer HJ was calculated to be ~7 D.

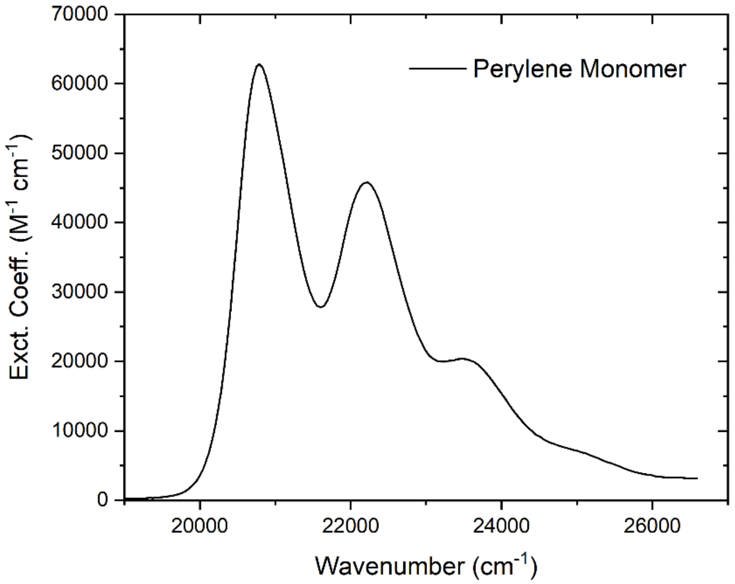

Figure S3. Extinction spectrum of DNA Holliday junction templated perylene monomer plotted as a function of wavenumber.

Section S3. Analysis of visible transient absorption of perylene monomer, transverse dimer, and adjacent dimer solutions

Global target analysis (GTA) was used to gain insight into the excited-state dynamics of the Holliday junction-templated perylene monomer as well as the transverse and adjacent perylene dimer solutions.

Monomer Solution

The TA of the monomer solution was fit according to a one-component kinetic scheme. An overlay of the data, the fit, and the residual at selected wavelengths of ~525 and 725 nm are shown in **Figure S4**. The analysis derived a time constant > 1.5 ns, which is the longest time delay measurable on the femtosecond visible transient absorption spectrometer.

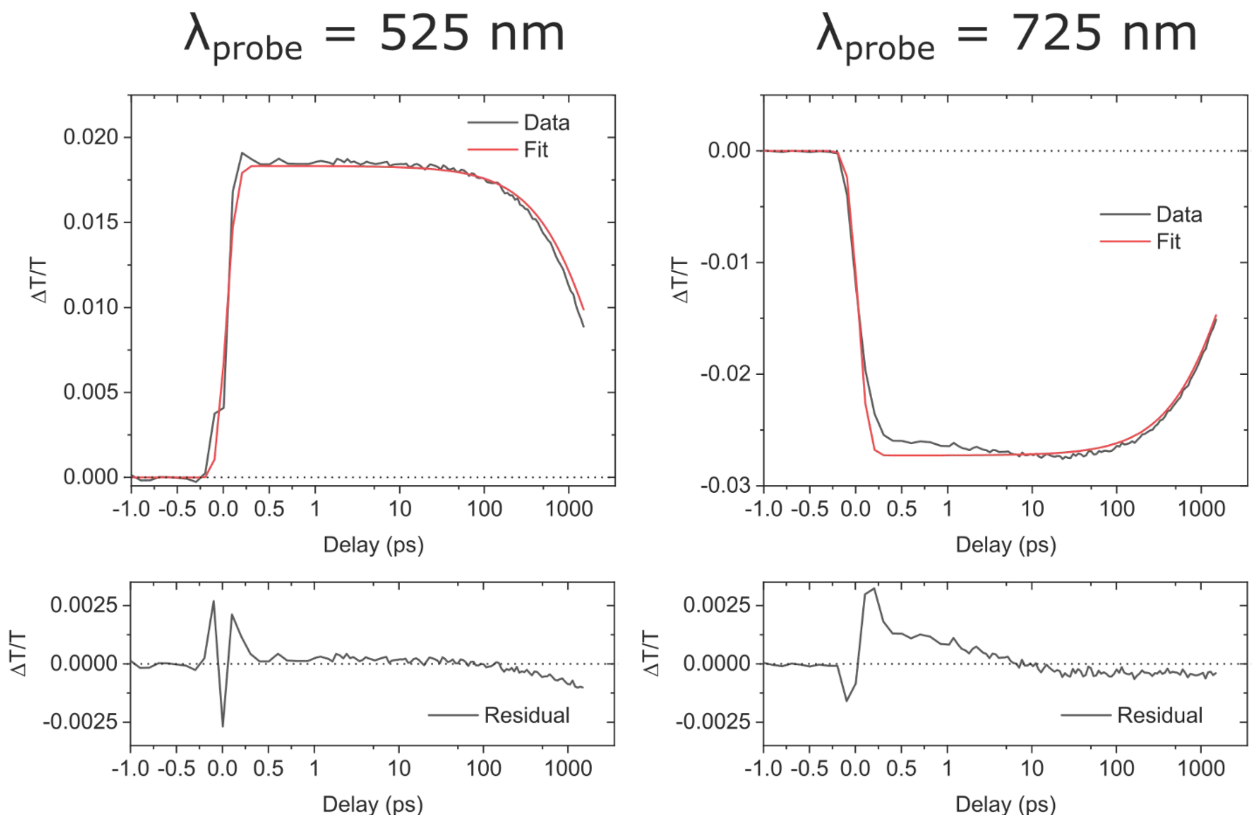

Figure S4. Results of a one-component global target analysis of the perylene monomer. Displayed are selected kinetic traces (black) and fits (red) of the most prominent SE and ESA bands taken at 525 and 725 nm, respectively. The 525 nm kinetic trace and fit is shown on the left with the residual plotted below. The 725 nm kinetic trace and fit is shown on the right with the residual plotted below.

Adjacent Dimer Solution

The TA of the perylene adjacent dimer solution was initially fit according to a two-component kinetic scheme. The basis for starting with a two-component kinetic scheme is as follows. Visual inspection of the decay kinetics at 600 nm, which are associated with an excited-state absorption (ESA) band, exhibit both growth and decay components. This is evident in the rise of the signal and its subsequent decay. **Figure S5** displays an overlay of the data, the fit, and the residual at selected wavelengths of ~600 and 525 nm. The analysis yields two time constants of ~50 ps and > 1.5 ns. As discussed in the main text, we attribute the ~50 ps time constant to perylene dimers forming non-emissive cations and anions due to symmetry breaking charge transfer (SB CT). We assign the > 1.5 ns time constant to the relaxation of the cation and anions in combination with relaxation of a subpopulation of perylene monomers decaying in parallel. While the fits to both 600 and 525 nm kinetics traces capture the essential behavior (i.e., the growth and decay of these signals, respectively, along with their subsequent decay), clearly the fit does not model the data well. Additionally, this is also evident in the structure present in the residuals.

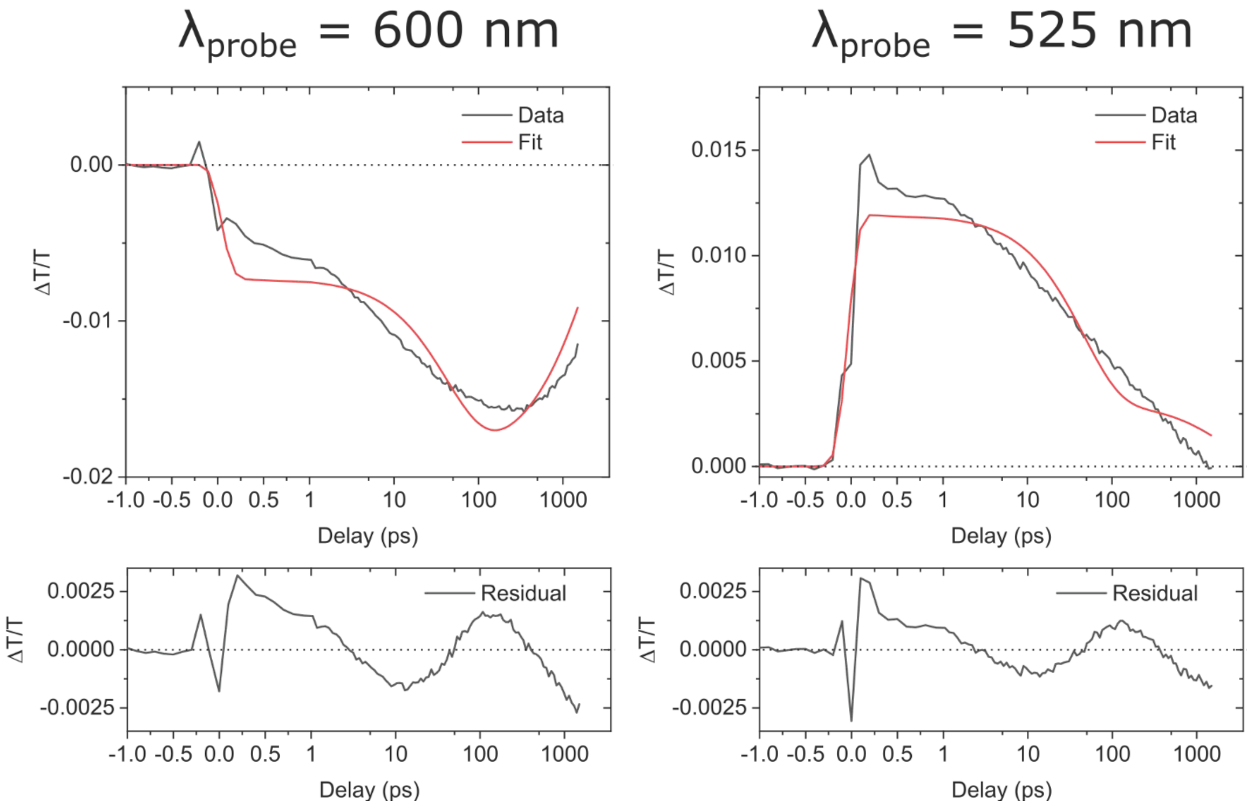

Figure S5. Results of two-component global target analyses of the perylene adjacent dimer. Displayed are selected kinetics traces (black) and fits (red) of the most prominent SE at 525 nm (right) and the ESA at 600 nm (left) that grows in over time.

Given that two components were unable to fully describe the kinetic behavior of the adjacent perylene dimer solution, GTA was performed with the addition of a third component. **Figure S6** displays an overlay of the data, the fit, and the residual at selected wavelengths of ~600 and 525 nm. The analysis yields three time constants of ~9.7 ps, ~300 ps, and > 1.5 ns. In the case

of the three-component kinetic scheme, **Figure S6** shows that the fit models the data quite well for both the 600 and 525 nm kinetic traces. Additionally, there is minimal structure in the residuals.

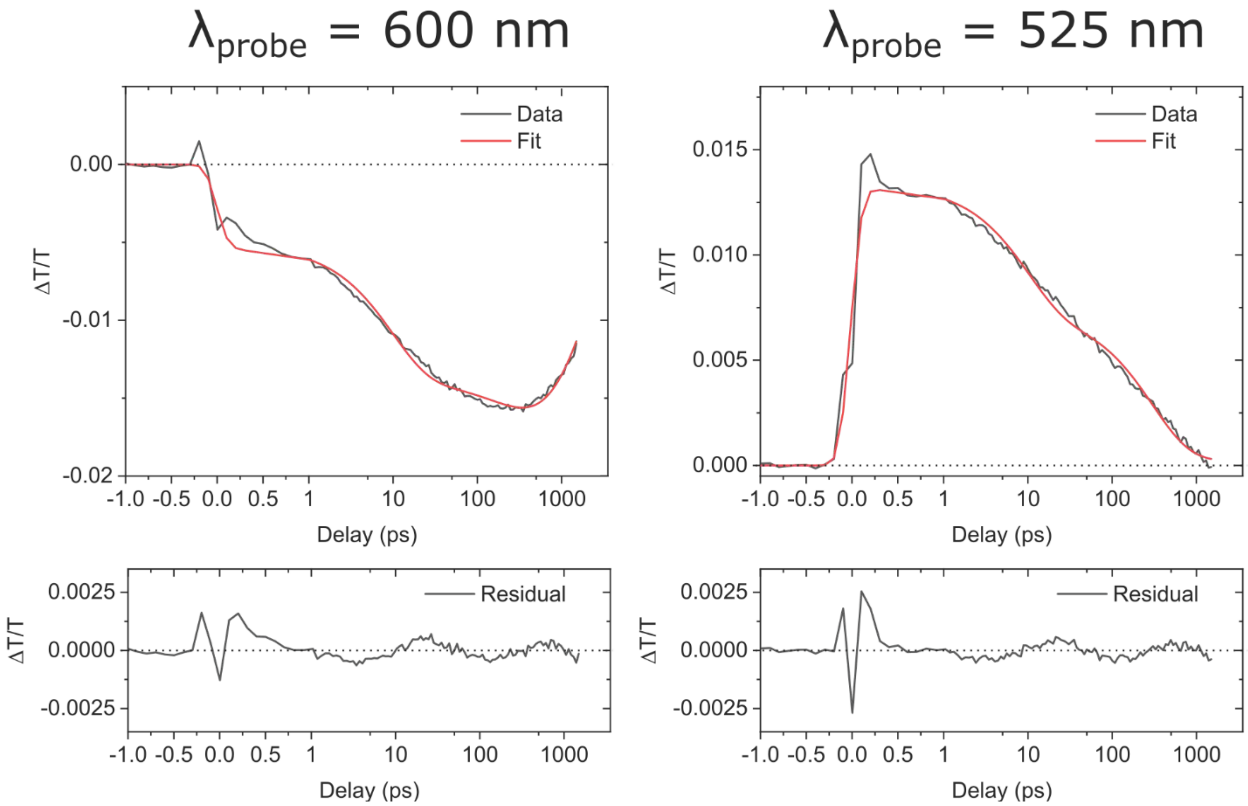

Figure S6. Results of three-component global target analyses of the perylene adjacent dimer. Displayed are selected kinetics traces (black) and fits (red) of the most prominent SE at 525 nm (right) and the ESA at 600 nm (left) that grows in over time.

We next turn to physical assignment of each component. We assign the 9.7 and 300 ps components to perylene adjacent dimers undergoing SB CT. That SB CT would take place over the course of two characteristic timescales is not unprecedented. For example, Markovic *et al.* (*Angew. Chem. Int.* **2011**, 50, 7596) synthesized propyl-linked perylene dimers and found SB CT took place in these dimers on characteristic timescales of about ~10 and ~100 ps. These authors explained the presence of the two characteristic timescales as resulting from a distribution of conformational states that the propyl-linked perylene dimers occupied. In DNA-templated molecular aggregates, similar structural heterogeneity has been observed (see e.g. Barclay *et al. Commun. Chem.* **2021**, 4, 19 and Huff *et al. J. Phys. Chem. B* **2021**, 125, 10240) that has been attributed to a distribution of distances between the dyes in the aggregates (see e.g. Huff *et al. J. Phys. Chem. B* **2021**, 125, 10240). Thus, we attribute the presence of the two characteristic timescales in the DNA-templated perylene adjacent dimers to a distribution of distances between perylenes. Finally, we assign the third >1.5 ns time constant, as above, to the relaxation of the cation and anions in combination with relaxation of a subpopulation of perylene monomers decaying in parallel.

To gain insight into the relative contribution of the 9.7 and 300 ps components to the ESA signal associated with SB CT, we fit the normalized 600 nm kinetics trace with a biexponential

growth function. We constrained the time constants to 9.7 and 300 ps, as these were the values derived from the GTA. **Figure S7** displays the 600 nm kinetics trace, which reaches its maximum amplitude by ~300 ps, along with the resulting biexponential growth function. The analysis yields an offset of 0.3 and relative amplitudes of 0.59 and 0.18 associated with the time constants 9.7 and 300 ps, respectively. As expected, the amplitude-weighted average time constant is ~80 ps, which is in good agreement with the 50 ps time constant derived by the two-component GTA. Thus, the present analysis shows that the two-component GTA effectively derives the average time constant associated with the two initial components of the three component GTA.

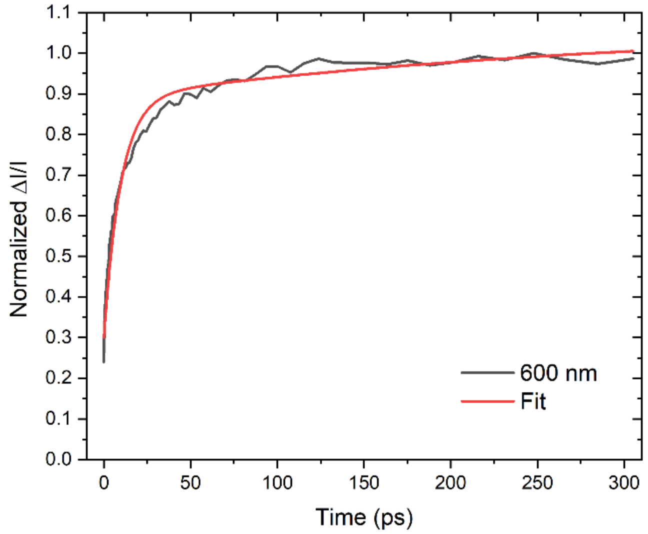

Figure S7. Result of biexponential fit of the growth function of adjacent dimer HJ at 600 nm. The time constants were constrained to those calculated through GTA to extract corresponding amplitudes.

Transverse Dimer Solution

As in the adjacent dimer, we fit the TA of the perylene transverse dimer solution with a two-component kinetic scheme. **Figure S8** displays an overlay of the data, the fit, and the residual at selected wavelengths of ~600 and 525 nm. The analysis yields two time constants of ~30 ps and > 1.5 ns. We attribute the ~30 ps time constant to perylene cations and anions resulting from SB CT and, as above, the > 1.5 ns time constant to the relaxation of the cation and anions in combination with relaxation of a subpopulation of perylene monomers decaying in parallel.

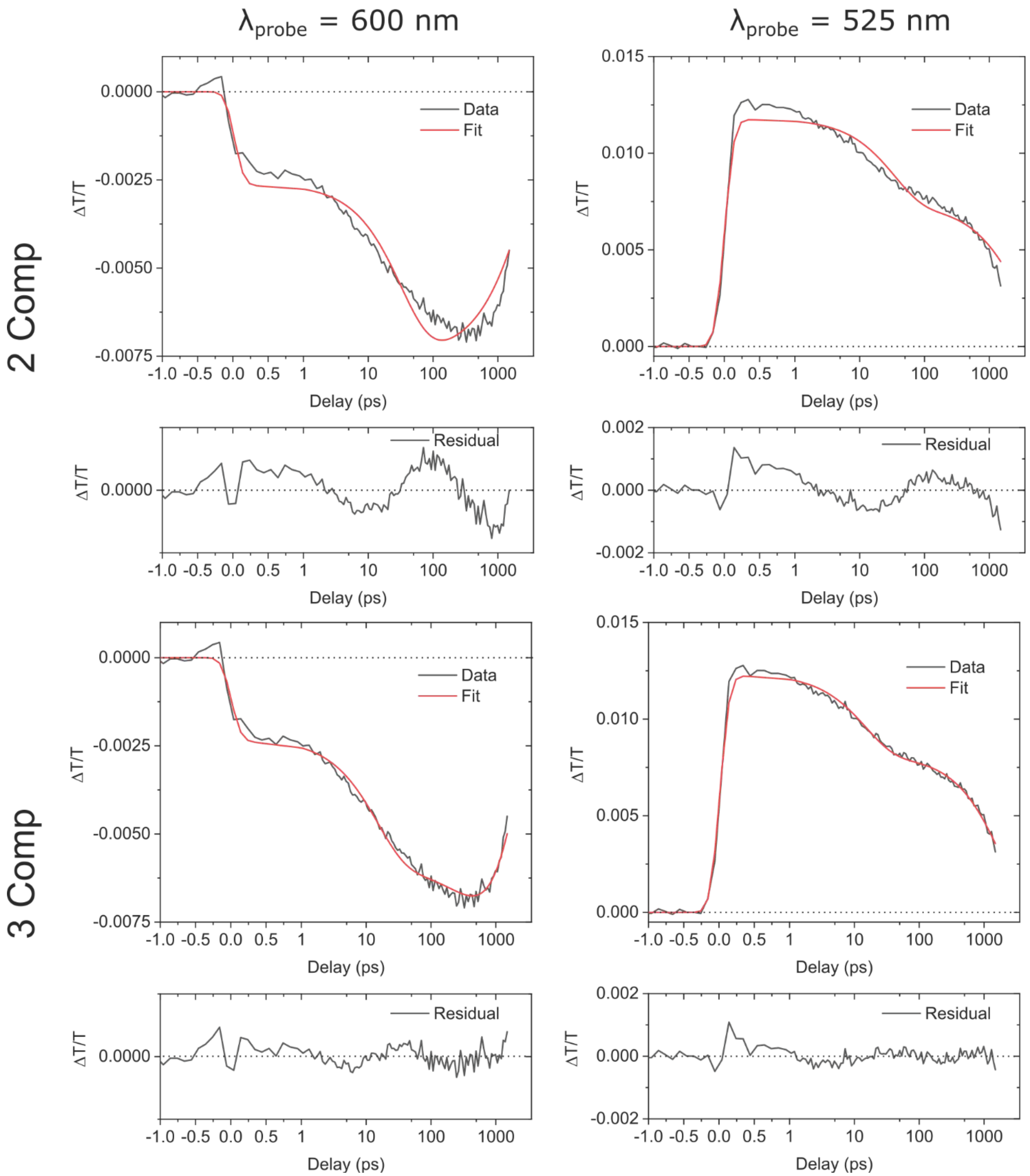

Figure S8. Results of two- and three-component global target analyses of the perylene transverse dimer. Displayed are selected kinetics traces (black) and fits (red) of the most prominent SE at 525 nm and the ESA at 600 nm that grows in over time. The left column shows 600 nm kinetics traces and fits for a two kinetic component (top) and three kinetic component (bottom) GTA with the fit residual plotted below. The right column shows 525 nm kinetics traces and fits for two kinetic component (top) and three kinetic component (bottom) GTA with the fit residual plotted below.

We also modelled the TA of the perylene transverse dimer solution using a three-component kinetic scheme. **Figure S8** displays an overlay of the data, the fit, and the residual at selected wavelengths of ~600 and 525 nm. The analysis yields three time constants of ~15 ps, 610 ps, and > 1.5 ns. As above, we attribute the first two time constants to perylene cations and anions resulting from SB CT, likely resulting from a distribution of distances between dyes within the dimer (see e.g. Markovic *et al. Angew. Chem. Int.* **2011**, 50, 7596 and Huff *et al. J. Phys. Chem. B* **2021**, 125, 10240). We assign the > 1.5 ns time constant to the relaxation of the cations and anions in combination with relaxation of a subpopulation of perylene monomers decaying in parallel.

Section S4. Nanosecond Visible Transient Absorption of Adjacent Perylene Dimer Solution

To further investigate the origin of the nonradiative decay observed in the perylene dimers, visible transient absorption (VIS TA) measurements were undertaken extending into the nanosecond timescale.

Figure S9 displays selected VIS TA spectra of solutions of the adjacent perylene dimer extending into the nanosecond timescale. On the earliest timescales (i.e., < 1 ps), negative-amplitude signals at 455 and 530 nm are observed and are attributed to ground state bleaching (GSB) and stimulated emission (SE), respectively. Another intense negative-amplitude peak is observed at 485 nm, which is attributed to a combination of GSB and SE. Additionally, positive-amplitude signals are observed above ~575 nm, which are attributed to excited-state absorption (ESA) features.

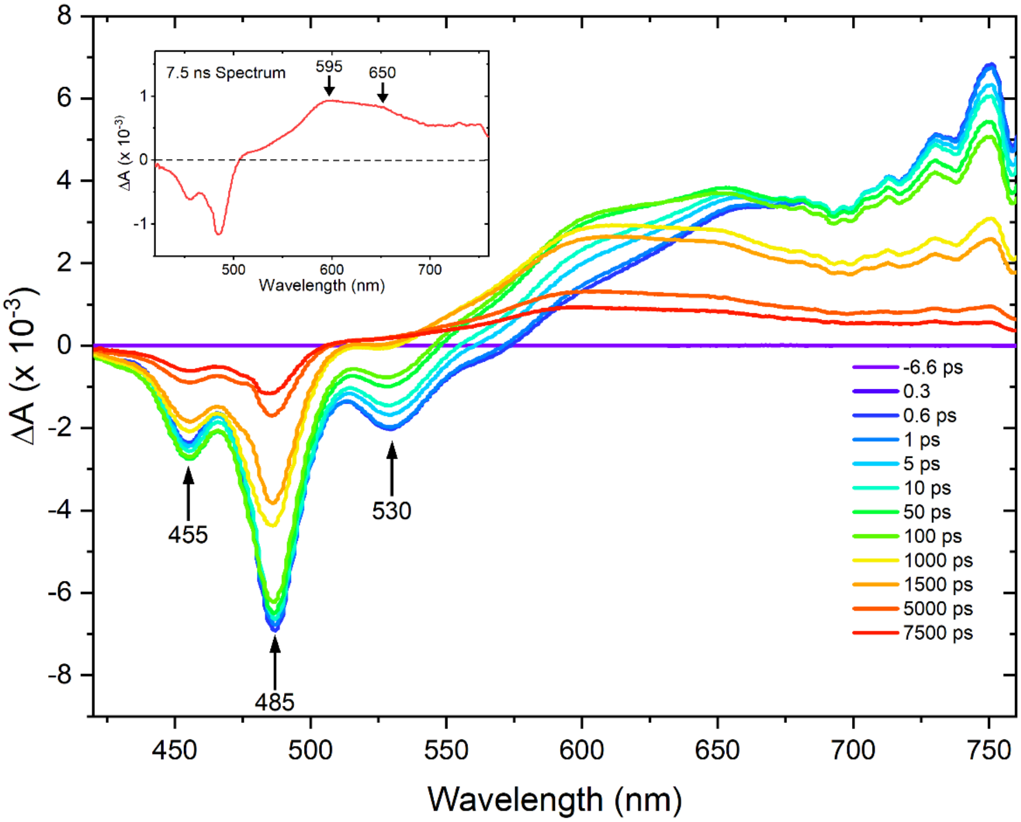

Figure S9. Selected spectra from VIS TA of DNA-templated perylene adjacent dimer solution excited at 480 nm. The specific wavelength of each peak is labeled with a black arrow pointing towards the peak. An inset of the spectra at 7500 ps is plotted in the top left corner with the specific peaks also labeled.

Interestingly, at long timescales (i.e., ≥ 5000 ps), the SE signal at 530 nm has decayed and ESA features at 595 and 650 nm remain (**Figure S9**, inset). These observations are consistent with the formation of perylene cations and anions, which are expected to be dark states.

Further, Markovic *et al.* (*Angew. Chem. Int.* **2011**, 50, 7596) showed that cations and anions form in propyl-linked perylene dimer dissolved in acetonitrile on a picosecond timescale via a process called symmetry-breaking charge transfer (SB CT). The perylene cations and anions exhibited two ESA peaks at ~540 and 585 nm, respectively. As discussed in more detail in the main text, if the ESA features at 595 and 650 nm observed in the DNA-templated perylene dimers are assigned to the cationic and anionic forms of the uracil-ethynyl-substituted perylene, equivalent redshifts of ~1,710 cm^{-1} are obtained in comparison with unsubstituted perylene. Specifically, calculating the redshift from 540 to 595 nm (i.e., cationic form) and from 585 to 650 nm (i.e., anionic form). Thus, the presence of two ESA features in the VIS TA on the $\geq$ 5 ns timescale along with their equivalent redshifts compared with the unsubstituted form of perylene support the interpretation that these ESA Features can be assigned to perylene cations and anions.
